# miR-200b-3p accelerates progression of pituitary adenomas by negatively regulating expression of RECK

**DOI:** 10.32604/or.2023.042581

**Published:** 2024-04-23

**Authors:** XIAOXI WANG, YANFEI JIA, QIANG LI, QIANG YANG, YINGFENG LIU, BEIFENG WEI, XIANG NIU, YINJIE ZHANG, XIAODONG LUO, ZIYU ZHAO, PENG WANG

**Affiliations:** 1 Department of Neurosurgery, Tianshui First People’s Hospital, Tianshui, China; 2 Department of Neurosurgery, The Second Affiliated Hospital of Lanzhou University, Lanzhou, China

**Keywords:** Pituitary adenomas, miR-200b-3p, RECK, Matrix metalloproteinase

## Abstract

MicroRNA (miR)-200b-3p has been associated with many tumors, but its involvement in pituitary adenoma is unclear. This study investigated the molecular mechanism underlying miR-200b-3p regulation in pituitary adenomas to provide a theoretical basis for treatment. Bioinformatics was used to analyze pituitary adenoma-related genes and screen new targets related to RECK and miRNA. As well, the relationship between miR-200b-3p and RECK protein was verified using a double-luciferase reporter gene assay. The expression of miR-200b-3p in clinical samples was analyzed by *in situ* hybridization. Transfection of the miR-200b-3p inhibitor and small interfering-RECK (si-RECK) was verified by qPCR. GH3 cell viability and proliferation were detected using CCK8 and EdU assays. Apoptosis was detected by flow cytometry and western blotting. Wound healing and Transwell assays were used to detect cell migration and invasion. The effects of miR-200b-3p and RECK on GH3 cells were verified using salvage experiments. miR-200b-3p was highly expressed in pituitary tumor tissue. Inhibitors of miR-200b-3p inhibited cell proliferation promoted cell apoptosis, inhibited invasion and migration, and inhibited the expression of matrix metalloproteinases. Interestingly, miR-200b-3p negatively regulated RECK. The expression of RECK in pituitary adenoma tissues was lower than that in neighboring tissues. Si-RECK rescued the function of miR-200b-3p inhibitors in the above cellular behaviors, and miR-200b-3p accelerated the development of pituitary adenoma by negatively regulating RECK expression. In summary, this study investigated the molecular mechanism by which miR-200b-3p regulates the progression of pituitary adenoma through the negative regulation of RECK. The findings provide a new target for the treatment of pituitary adenoma.

## Introduction

Pituitary adenomas (PAs) are common intracranial tumors that account for approximately 10% of intracranial tumors [1]. Growth hormone (GH) pituitary tumors are second only to nonfunctional adenomas and prolactinomas in the incidence of pituitary tumors [2]. Excessive secretion of GH directly or indirectly acts on various organs, leading to the dysfunction of multiple organs and systems [3]. Some tumors display infiltrating growth and relapse after surgery; drug intervention is very easy [4]. Despite numerous basic and clinical investigations of the relevant molecular mechanisms of pituitary tumors, the pathogenesis of most pituitary tumors remains controversial. Therefore, it is critical to study the pathogenesis of pituitary adenomas and explore the important genes involved.

MiR-200b-3p is a microRNA (miRNA) closely associated with human tumors. Many related studies have confirmed that miR-200b-3p can regulate tumor progression [5]. Zhou et al. found that miR-200b-3p is downregulated in melanoma tissues and cell lines and promotes melanoma progression by activating epithial-to-mesenchymal transition (EMT) [6]. Chi et al. described that miR-200b-3p affects the occurrence and development of lung cancer by negatively regulating the expression of large tumor suppressor kinase 2 (LATS2) and suppressor of cytokine signaling 6 (SOCS6) [7]. Li et al. demonstrated that miR-200b-3p inhibits the proliferation, migration, and invasion of breast cancer cells through the LIM domain kinase1/cofilin 1 (LIMK1/CFL1) pathway [8]. Chen et al. found that miR-200b-3p can inhibit the proliferation of colorectal cancer cells and induce apoptosis by inactivating Wnt/β-catenin signaling [9].

Reversion-inducing cysteine-rich protein with kazal motifs (RECK) is a membrane-anchored protein that regulates matrix metalloproteinases (MMPs) [10], which are involved in the invasion and metastasis of a variety of tumor cells and patient prognosis [11]. The latter study compared the expression of RECK in GH and non-GH pituitary tumors, explored its correlation with GH pituitary tumors, and investigated the effect of RECK on GH3 pituitary tumors and its related mechanisms. The findings provide theoretical support for the tumor-targeted treatment of GH pituitary tumors.

It has been reported that some small RNAs directly regulate gene switching and participate in growth and differentiation [12]. In addition, they are involved in cell differentiation and apoptosis by decreasing the stability and expression of target genes [13,14]. Therefore, exploring the relationship between miRNAs and the formation of pituitary tumors provides a novel method to identify the pathogenic factors of pituitary tumors. Bottoni et al. first discovered the connection between miRNAs and pituitary tumors in 2005 [15]. These authors confirmed that miR-15a and miR-16-1 were weakly expressed in PAs and negatively correlated with tumor diameter. In 2019, Zhang et al. published the findings of a genome-wide comparative analysis of invasive and non-invasive pituitary adenocarcinoma samples and screened various miRNAs as potential targets for treatment of PAs [16]. Although various miRNAs have been screened, their effective targets and detailed molecular mechanisms remain unclear.

Large-scale expressed sequence tag analysis has established the gene expression profiles of different pituitary tumor tissues. The comparison of these profiles with those of normal pituitary tissues will deepen our understanding of the pathogenesis of pituitary tumors. In this study, using bioinformatic database analysis, miR-200b-3p was identified as a feasible therapeutic target for PA, and its molecular mechanism was verified through cell experiments *in vitro*. The findings of this in-depth study of the molecular mechanisms provide theoretical support for the treatment of pituitary tumors.

## Materials and Methods

### Screening of potential targets

First, genes related to pituitary tumors were obtained by genome analysis (https://www.genecards.org/), followed by enrichment analysis (https://biit.cs.ut.ee/gprofiler/gost) g:profiler to obtain the most significant miRNAs. Second, RECK-related miRNAs were identified from the (http://starbase.sysu.edu.cn/index.php), Starbase (https://ngdc.cncb.ac.cn/databasecommons/database/id/3941), miRWALK, and (http://mirdb.org/) miRDB databases. These identified miRNAs and the miRNAs related to pituitary tumors enriched in g:profiler were analyzed using TargetScan for intersection analysis. The novelty of the miRNAs was determined using NCBI.

### Collection of patients’ tissues

Pituitary adenoma and adjacent tissues were obtained from surgically resected specimens of 12 patients at our hospital. The included patients had not been treated with radiotherapy or chemotherapy. The specimens were washed with normal saline, snap-frozen, and kept at −80°C. This study was approved by the ethics committee of Tianshui First People’s Hospital (Approved ID: 2022003), and the patients were informed and agreed.

### In situ hybridization (ISH) assay

The pre-frozen tissue blocks were moved into a cryostat microtome for sectioning at a thickness of 10 μm. The sections were adhered to gelatin-treated glass slides, rapidly dried, fixed for 20 min with 40 g/L paraformaldehyde, washed with PBS (0.1 mol/L), treated with a pre-hybridization solution, dehydrated, and degreased with ethanol and chloroform. During hybridization, 200 μL of hybridization solution, 15 μL dithiotreitol (DTT), and labeled probes were dropped onto the surface of the sections and incubated in a humid environment for 24 h. After hybridization, the slices were washed with 1× SSC, dehydrated, and dried. Nuclear latex was then applied to the surface of the sections, which were placed in a dark box for exposure. Finally, each sample was developed using Kodak D19 developer, fixed with F-5 hard film fixer, washed with water, counterstained with thionine, dehydrated, made transparent, sealed with neutral gum, and observed using light microscopy in both light and dark fields.

### GH3 culture and treatment

GH3 rat pituitary tumor cells (American Tissue Culture Collection, Manassa, VA, USA) were cultured using F-12K medium containing 15%, 2.5% fetal bovine serum, and 1% penicillin/streptomycin, incubated at 37°C, with 5% CO_2_. The cells were then digested with 0.25% trypsin and used for experiments after passage.

miR-200b-3p inhibitor, small interfering-RECK (si-RECK), and their controls were designed and synthesized by Biomics Biotechnologies Co., Ltd. (Nantong, Jiangsu, China). When the growth of cells plated on a 6-well plate was more than 60%, and the inhibitors (NC) and miR-200b-3p were added with serum-free medium and X-tremeGENE transfection reagent (Roche, USA) and incubated for 20 to 30 min. Total RNA and protein were extracted after 24 h of culture.

For the rescue assay, cells were transfected with si-RECK simultaneously. A total of 4 μL si-RECK and its control were mixed with 200 μL Opti-DMEM for 5 min. At the same time, 4 μL Liposome 2000 was mixed with 200 μL Opti-DMEM for 5 min. These solutions were blended and incubated for 20 min at 37°C on a clean bench. Transfection was performed as described previously.

### Quantitative real-time reverse-transcription PCR assay

The primers designed using Primer Premier software (version 6.0) according to the gene sequences in GenBank were synthesized by the Shanghai Shenggong Company. The total qRT-PCR reaction system was 30 μL, including 2 μL of cDNA, 1 μL of the upstream and downstream primers (50 pmol/μL), 12.5 μL of 2× TransStart™ Top Green PCR mixture, and RNase free double-distilled water to produce the final volume. The reaction conditions included an initial denaturation at 94°C for 2 min, 40 cycles of 94°C for 30 s, 58°C for 30 s, and 72°C for 30 s, and final extension at 72°C for XX. The primers used were as follows: miR-200b-3p, (F) 5′-GTGCAGGGTCCGAGGT-3′, (R) 5′-UAAUACUGCCUGGUAAUGAU-3′; RECK, (F) 5′-GCAGGGGAAGTTGGTTGTTA-3′, (R) 5′-TGCCAGCAAAACAAGAACAG-3′. The Light Cycler 480 fluorescence quantitative PCR instrument software was used to collect fluorescence signals at 72°C and perform curve analysis. The results were calculated using the 2^−ΔΔCt^ method.

### CCK8 assay

A total of 1 × 10^6^/mL GH3 cells from each group were seeded into 96-well plates (0.1 mL per well). After 12 h of culture, 0.1 mL of the culture medium containing 10% CCK8 reagent was replaced. The cells were incubated at 37°C, protected from light, for 2 h. The optical density at 450 nm (OD450) was detected using a model ELx800 automatic microplate reader (BioTek Instruments, USA).

### 5-Ethynyl-2′-deoxyuridine (EdU) experiment for cell proliferation

GH3 cells (4 × 10^3^/mL) from each group were seeded in the wells of 96-well plates. After 12 h, 0.1 mL of EdU-containing medium was added and incubated for 2 h. Cells fixed with paraformaldehyde were permeabilized with Triton X-100, followed by the addition of 0.1 mL of Apollo staining reaction solution. After mounting the slides, cell proliferation was measured using the aforementioned ELx800 automatic microplate reader.

### Flow cytometry detection of apoptosis

After digestion, centrifugation, and washing of each group of GH3 cells, binding buffer (100 μL) and Anexin-V-FITC (20 ng/L, 10 μL) were sequentially added. The samples were incubated at 37°C for 0.5 h in the dark. Propidium iodide reagent (50 ng/L, 5 μL) was added followed by incubation at 37°C for 5 min in the dark. Finally, 0.4 mL of the binding buffer was added, and the rate of apoptosis was quickly detected using a FACSCanto II flow cytometer (BD Biosciences, USA).

### Western blot detection of apoptosis and matrix metalloproteinase (MMP)-related proteins

Protease and phosphatase inhibitors were added to the RIPA lysis buffer to prepare the protein lysis buffer. Cell lysate was added to the treated GH3 cells. The cells were scraped off the surface of the 6-well plate and centrifuged at 4°C and 12,000 r/min for 10 min to extract the total cell protein. The protein concentration was determined, and samples with equal protein content were aliquoted and stored at −80°C. A total of 40 μg total protein lysate was added to a 10% SDS-PAGE gel. Proteins were concentrated and separated at 60 and 100 V, respectively. The resolved proteins were transferred to PVDF membranes at 100 V for 75 min. The membranes were blocked with 10% skimmed milk powder for 2 h at 37°C. Membranes were then exposed to primary antibodies (1:1000 dilution each). The antibodies used were: anti-Bax, ab32503; Bcl2, ab32124; anti-cleaved-caspase 3, E83-77; anti-RECK, ab89915; anti-MMP2, ab92536; anti-MMP9, ab76003; and anti-ß-actin, ab8226 (all from Abcam, USA). After incubating overnight at 4°C, secondary antibody (1:50,000, Thermo Fisher Scientific, USA) was added and incubated at 37°C for 1 h. The membranes were exposed to X-ray film through the ECL detection system.

### Wound healing assay

Aliquots (1 mL) of a cell suspension (1 × 10^9^ cells/L) were seeded in 6-well plates. Serum-free DMEM was added to the culture for 6 h to generate a monolayer of adherent cells. A scratch was made in the monolayer using a sterile 10 μL Eppendorf tip. The scratched monolayer was washed three times with serum-free medium, and more serum-free medium was added. The cells were incubated according to groups at 37°C and 5% CO_2_ for 24 h. The cells were photographed at 0 and 24 h, and the scratch distance was measured using Image-Pro Plus 6.0. The experiment was performed three times, and cell migration ability was calculated based on the scratch width.

### Transwell assay for migration and invasion assay

*In vitro* cell invasion experiments used a 24-hole Transwell plate with a pore size of 8.0 μm. The cells were treated according to the groups described above. Matrigel frozen at −80°C was stored at 4°C overnight to liquify. Matrigel was diluted 1:9 with serum-free medium, and 50 μL was evenly spread on the upper chamber of the Transwell chamber and incubated overnight at 37°C. A total of 100 μL pre-warmed, serum-free DMEM was added to each upper chamber and incubated at room temperature for 30 min to rehydrate the matrigel. The remaining culture medium was removed, and the cells were digested and washed three times with serum-free medium. One hundred microliters of cell suspension containing 1 × 10^9^ cells was added to the upper chamber, and 500 μL of complete medium was added to the lower chamber. After incubating at 37°C in an atmosphere of 5% CO_2_ for 24 h, the medium was discarded and the surface of each upper chamber cell was gently wiped with a cotton swab. The Transwell chamber was washed twice with PBS, and the cells were fixed with 4% paraformaldehyde for 10 min, inverted, and air-dried. After staining with a 0.1% crystal violet solution for 20 min, the cells were counted using inverted microscopy. Matrigel was not used in the cell migration assay.

### Dual-luciferase reporter gene assay

The 3′ untranslated region (3′UTR) of the miR-200b-3p wild-type (wt) target region and its mutant were inserted into the RECK vector to construct the RECK-wt and RECK-Mut plasmids, respectively. The transfected cells were divided into four groups: miR-200b-3p mimic+RECK-wt, miR-200b-3p mimic+RECK-mut, NC mimic+RECK-wt, and NC mimic+RECK-mut. The final concentration used for each group was 20 nM. Each group was cultured in the corresponding cell culture medium for 6 h after the medium was replaced. After 24 h of transfection, cells were lysed, and 10 μL of supernatant was obtained. One hundred microliters of Reagent II was added to each well of a 96-well cell culture plate. After 5 s, luciferase activity was measured by enzyme-linked immunosorbent assay.

### Statistical analysis

SPSS 19. 0 software and GraphPad Prism 6.0 were used for statistical analyses. Data are expressed as mean ±standard error. An independent sample *t*-test was used to compare the two groups. Single-factor analysis of variance was performed for multiple group comparisons, and the Student-Newman-Keuls method was used for multiple comparisons. The enumeration data used the χ^2^ test. Statistical significance was set at *p* < 0.05.

## Results

### Higher expression of miR-200b-3p in PA

**Figure 1:**
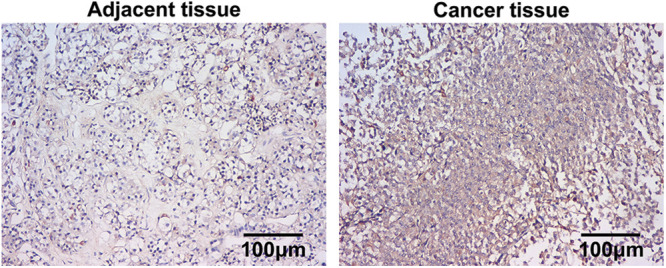
Higher expression of miR-200b-3p in pituitary adenomas. Scale bar = 100 μm.

*In situ* hybridization (ISH) was performed to determine the level of miR-200b-3p in clinical tissues. The results confirmed that adenocarcinoma samples expressed miR-200b-3p more than the adjacent tissues (Fig. 1).

### miR-200b-3p inhibitor suppresses cell proliferation and enhances apoptosis

**Figure 2:**
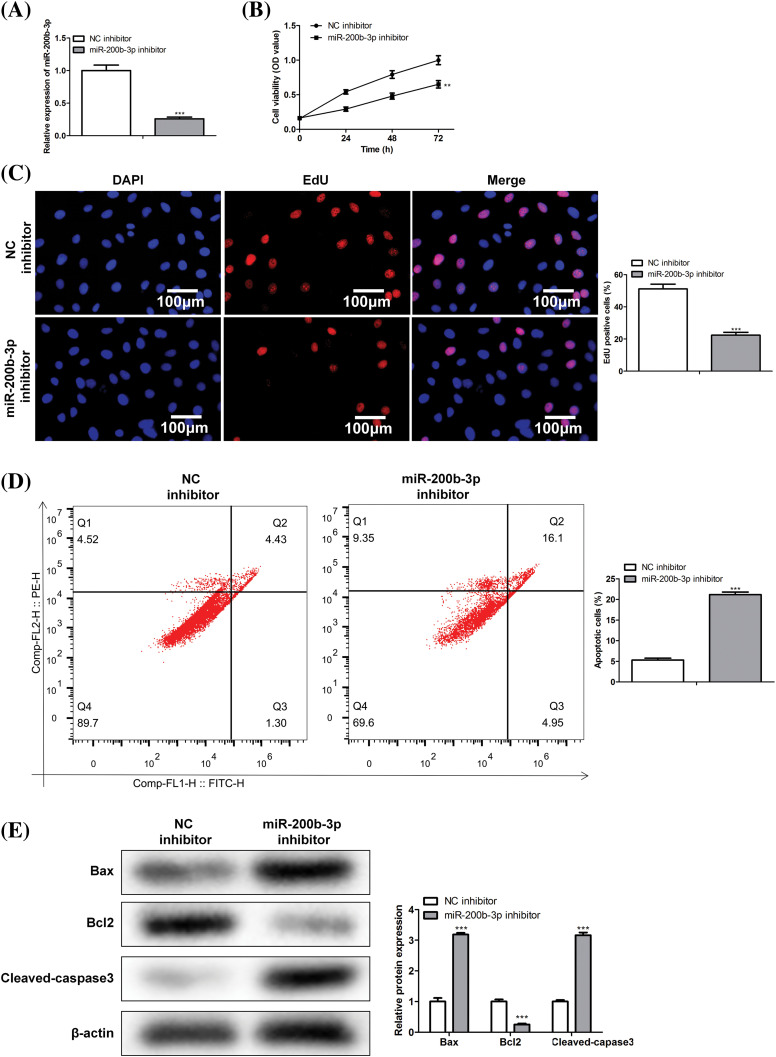
miR-200b-3p inhibitor suppresses cell proliferation ability and improves apoptosis. (A) The level of miR-200b-3p was significantly suppressed by miR-200b-3p inhibitor. Suppressed cell activity (B) and cell proliferation (C) by miR-200b-3p inhibitor. Scale bar = 100 μm. (D) Apoptosis of GH3 cells is enhanced by miR-200b-3p inhibitor. (E) Enhanced Bax and cleaved-caspase 3 levels and significantly lower Bcl2 levels after miR-200b-3p transfection. **p < 0.01, ***p < 0.001.

Cells were divided into NC and miR-200b-3p inhibitor groups to study the effect of miR-200b-3p on the viability of PA cells. Inhibition of miR-200b-3p suppressed miR-200b-3p levels, indicating successful transfection (Fig. 2A). Moreover, cell activity and proliferation were negatively affected (Figs. 2B and 2C). Importantly, the miR-200b-3p inhibitor accelerated GH3 cell apoptosis (Fig. 2D). The levels of apoptosis-related proteins remained consistent. Bax and cleaved-caspase 3 levels were clearly higher, whereas Bcl2 levels decreased after miR-200b-3p transfection (Fig. 2E).

### miR-200b-3p inhibitor suppressed invasion and migration, and inhibited the expression of matrix metalloproteinase

**Figure 3:**
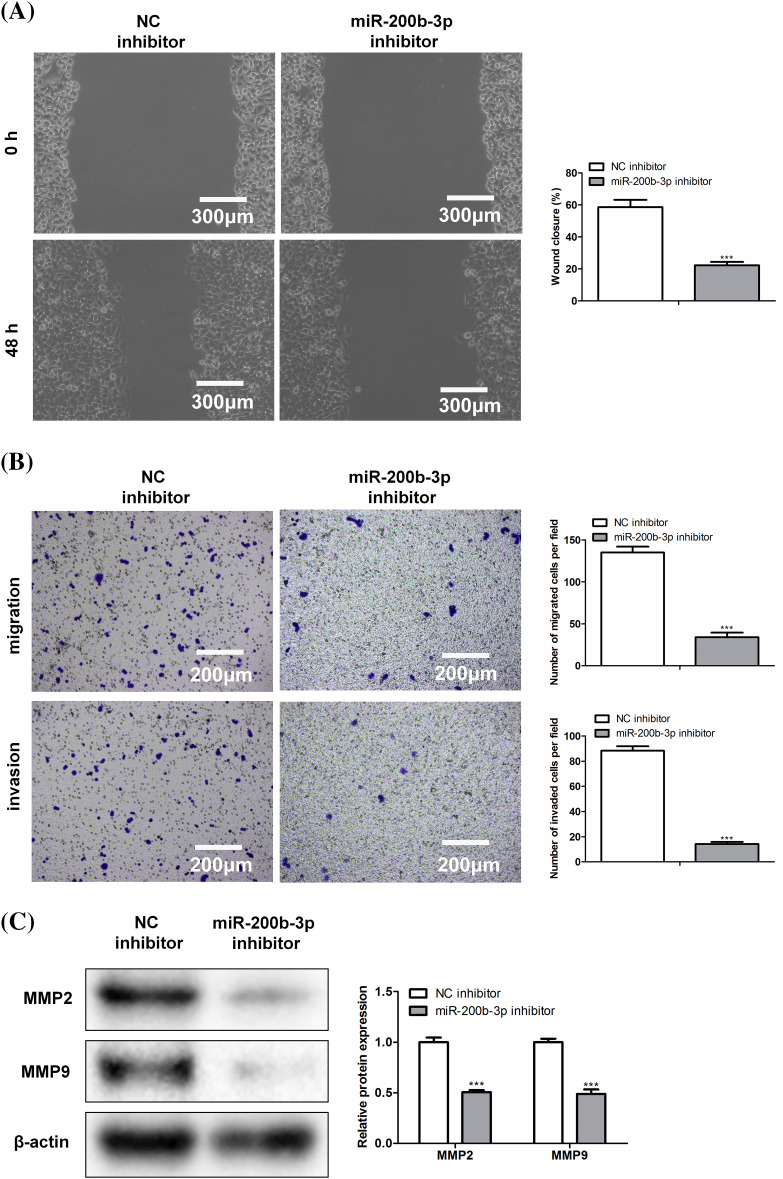
Inhibition of miR-200b-3p suppresses invasion and migration, and inhibits the expression of matrix metalloproteinase (MMP). (A) Compared with NC inhibitor group, scratch width was larger in miR-200b-3p inhibitor group. Scale bar = 300 μm. (B) Inhibition of miR-200b-3p repressed migration and invasion of GH3 cells. Scale bar = 200 μm. (C) MMP2 and MMP9 expressions were lower in miR-200b-3p inhibitor group compared to the levels in control cells. ***p < 0.001.

The scratch width was greater in the miR-200b-3p inhibitor group than in the NC inhibitor group (Fig. 3A). Moreover, miR-200b-3p inhibitors inhibited GH3 cell migration and invasion (Fig. 3B). Matrix metalloproteinase levels were determined using western blotting. Fig. 3C showed both MMP2 and MMP9 were expressed at lower levels in the miR-200b-3p inhibitor group than in the control.

### Negative regulation of RECK by miR-200b-3p

**Figure 4:**
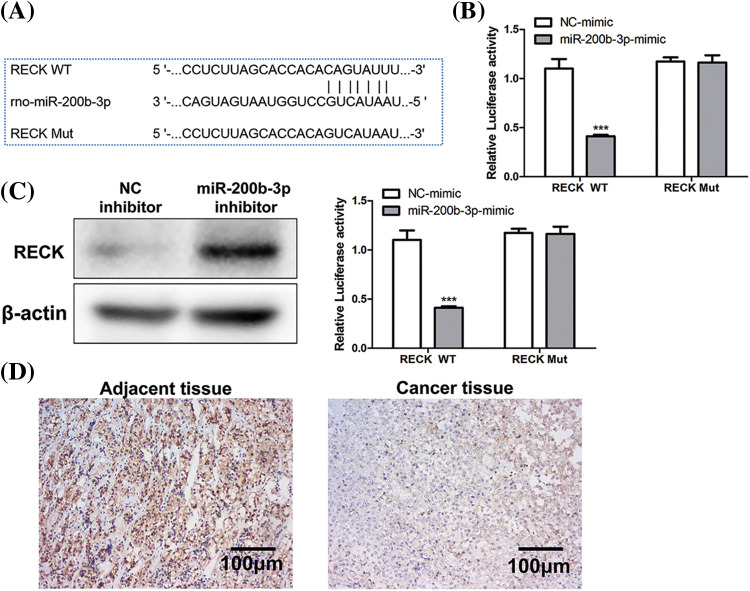
Negative regulation of RECK by miR-200b-3p. (A) The binding site between miR-200b-3p and RECK. (B) Dual-luciferase reporter gene assay detection of binding. (C) Inhibition of miR-200b-3p accelerates RECK expression in GH3 cells. (D) Significantly lower RECK expression in PA tissue compared to expression in adjacent tissue. Scale bar = 100 μm. ***p < 0.001.

The binding site between miR-200b-3p and RECK is shown in Fig. 4A. A dual-luciferase reporter gene experiment was performed to detect binding. In RECK mutant cell lines, transfection with the miR-200b-3p mimic had no effect on RECK expression. However, RECK expression was downregulated in the RECK-wt cells (Fig. 4B). Western blot was performed to detect expression relationships at the protein level. The miR-200b-3p inhibitor increased RECK expression in GH3 cells (Fig. 4C). Importantly, clinical samples were processed for immunohistochemistry (IHC) to detect RECK expression. RECK expression was lower in PA tissue than in the adjacent tissue (Fig. 4D).

### si-RECK rescues the effect of miR-200b-3p inhibitor

A rescue assay was performed to confirm the effects of miR-200b-3p and RECK on GH3 cells. The cells were divided into three groups: NC inhibitor + si-NC, miR-200b-3p inhibitor + si-NC, and miR-200b-3p inhibitor + si-RECK.

**Figure 5:**
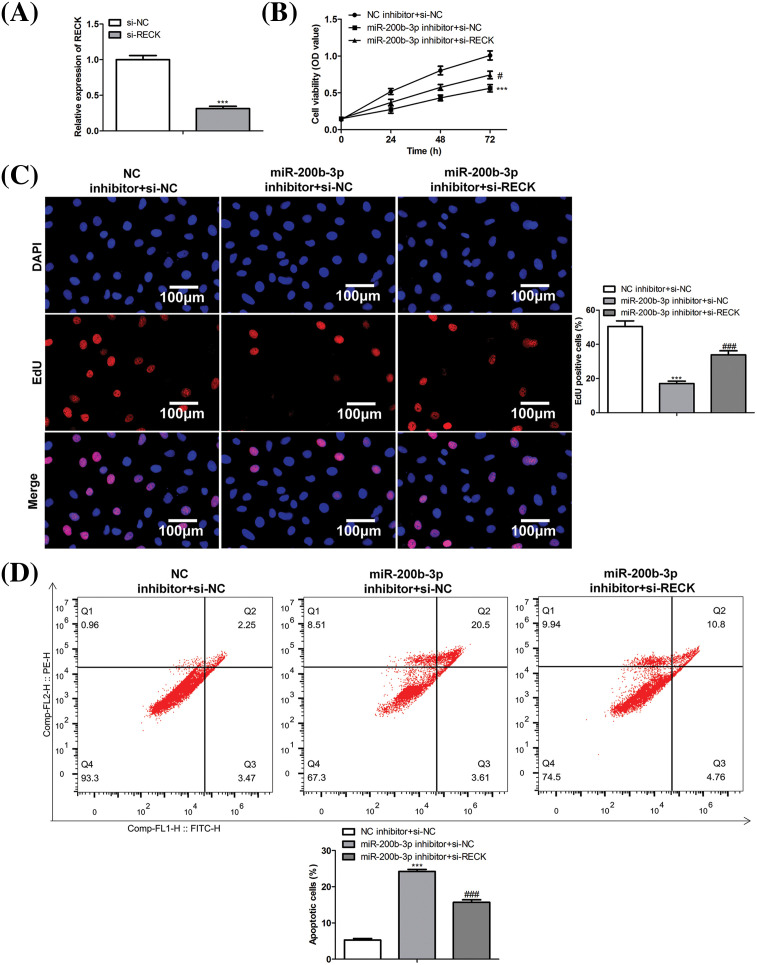

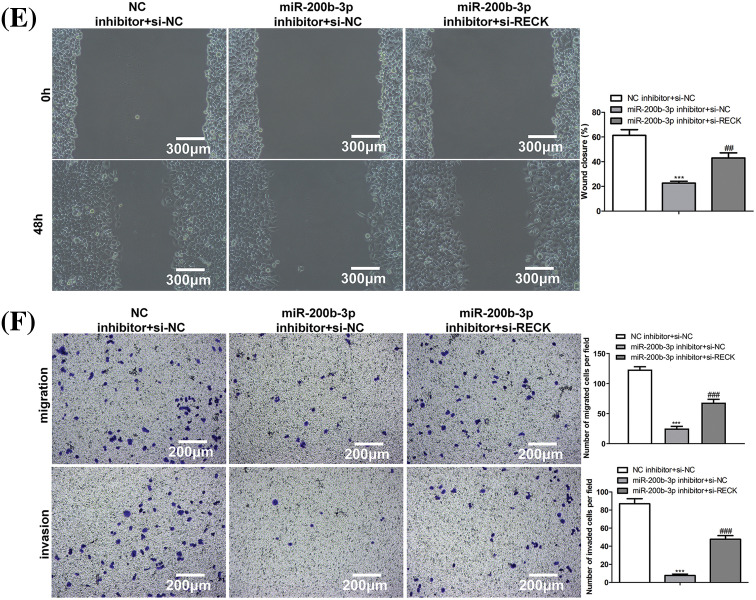
si-RECK rescues the effect of miR-200b-3p inhibitor. (A) si-RECK transfection down-regulates RECK expression in GH3 cells. (B and C) Inhibition of miR-200b-3p suppresses cell proliferation, while si-RECK rescues the effect of miR-200b-3p inhibitor. Scale bar = 100 μm. (D) Inhibition of miR-200b-3p accelerates apoptosis, while si-RECK rescues this effect. (E) Si-RECK reduced the scratch width compared with miR-200b-3p inhibitor groups. Scale bar = 300 μm. (F) miR-200b-3p inhibitor suppressed invasion and migration of GH3 cells, while si-RECK rescued the effect. Scale bar = 200 μm. ***p < 0.001, ^#^*p* < 0.05, ^##^*p* < 0.01, ^###^*p* < 0.001.

After si-RECK transfection, RECK expression decreased in GH3 cells, indicating successful transfection (Fig. 5A). Inhibition of miR-200b-3p suppressed cell proliferation, whereas si-RECK rescued the effects of the miR-200b-3p inhibitor (Figs. 5B and 5C). Moreover, the miR-200b-3p inhibitor accelerated apoptosis and suppressed the invasion and migration of GH3 cells. Interestingly, si-RECK rescued the function of miR-200b-3p in these cell behaviors (Figs. 5D–5F).

## Discussion

The occurrence of PA is related to many factors, such as environment, behavior, and genetics [17]. The etiology of 95% of PAs is unknown. Finding new specific molecules as therapeutic targets is a hot topic in PA research. In this study, miR-200b-3p, screened by a bioinformatics database, was expressed at higher levels in PA tissue. Inhibition of miR-200b-3p suppressed invasion and migration and enhanced apoptosis of GH3 cells.

miR-200b-3p is involved in various physiological activities in the body [18,19]. These include the regulation of monocyte and macrophage differentiation [20]. Additionally, miR-200b-3p has been associated with breast, prostate, and colorectal cancers, oral squamous cell carcinoma, and gliomas [8,21]. miR-200b also affects the growth of intestinal epithelial cells in inflammatory bowel disease (IBD). Its expression in the mucosa of IBD lesions is reduced [22]. Therefore, a high expression of miR-200b-3p in PA was expected.

Inhibition of miR-200b-3p suppressed MMP expression. MMPs are proteolytic enzymes that destroy connective tissue and basement membranes; these enzymes are crucial in tumor invasion [23]. MMP-2 and MMP-9 are the two most widely studied invasive factors. Nakada et al. confirmed that the expression of MMP-2 in glioblastoma multiforme was higher than that in astrocytoma, and similar positive results were found in the cerebrospinal fluid of patients [24]. Pereda et al. investigated the relationship between the function and expression of MMPs in pituitary tumor cells. The researchers described how high levels of MMP activity regulated tumor proliferation and hormone secretion [25]. Both MMP-9 and MMP-2 belong to a subfamily of gelatinases that enzymatically hydrolyze basement membrane IV collagen [26]. Collagen IV is a key component of the dura mater that protects against tumors and inflammatory invasion. In seven cases of PA, Kawamoto et al. found that MMP-9 was positively expressed in all invasive tumor cells, and the collagenase IV activity level in invasive PA cells and the dura mater was significantly higher than that in non-invasive PAs [27]. These findings indicate that collagenase IV activity is closely associated with PA invasiveness. Tomita et al. performed an immunochemical assay and found that tissue inhibitor of metalloproteinases 1 in PAs MMP-2, MMP-9, and normal anterior pituitary cells showed positive staining [28]. MMP-9 is associated with the invasion, recurrence, and angiogenesis of PAs. Liu et al. proposed injecting synthetic MMP-9 inhibitors to treat PAs [29]. Similar results were obtained in this study. Inhibition of miR-200b-3p inhibitor suppressed PA development by inhibiting MMP-2 and MMP-9 expression.

Interestingly, miR-200b-3p negatively regulates RECK expression. In 1998, Takahashi et al. expressed a new type of tumor suppressor gene (RECK) isolated from cDNA in mouse MH3T3 cells transfected with the v-Ki-ras gene; the size of the expressed protein was 110 kDa [30]. RECK is a membrane-anchored protein that regulates MMPs, which are closely associated with the invasion and metastasis of various tumor cells and patient prognosis [31]. RECK inhibits the activity of MT1-MMP, MMP-2, and MMP-9 [32]. In addition, RECK expression is markedly aberrant in many different tumors, including lung, bladder, breast, and pancreatic cancer [33,34]. Patients with high RECK expression have a significantly better prognosis than those with low RECK expression; with decreased or absent RECK expression, cells undergo malignant transformation [35]. In this study, si-RECK rescued the function of the miR-200b-3p inhibitor in PA cells, confirming that miR-200b-3p accelerates the development of PA by negatively regulating RECK levels.

The limitations of this study are that we only studied GH3 cells due to economic issues and did not perform animal experiments. If animal experimental validation was included, the data would be more credible. This will be the focus of our future study.

## Conclusions

PAs are accelerated by the negative regulation of RECK expression through miR-200b-3p. This study identifies a novel target for the treatment of pituitary adenomas.

## Data Availability

Readers can access the data used in the study from corresponding author.
